# Genome-Wide Identification of Polycomb Target Genes Reveals a Functional Association of Pho with Scm in *Bombyx mori*

**DOI:** 10.1371/journal.pone.0034330

**Published:** 2012-04-02

**Authors:** Zhiqing Li, Daojun Cheng, Hiroaki Mon, Tsuneyuki Tatsuke, Li Zhu, Jian Xu, Jae Man Lee, Qingyou Xia, Takahiro Kusakabe

**Affiliations:** 1 Laboratory of Silkworm Science, Kyushu University Graduate School of Bioresource and Bioenvironmental Sciences, Fukuoka, Japan; 2 State Key Laboratory of Silkworm Genome Biology, Southwest University, Chongqing, China; University of Georgia, United States of America

## Abstract

Polycomb group (PcG) proteins are evolutionarily conserved chromatin modifiers and act together in three multimeric complexes, Polycomb repressive complex 1 (PRC1), Polycomb repressive complex 2 (PRC2), and Pleiohomeotic repressive complex (PhoRC), to repress transcription of the target genes. Here, we identified Polycomb target genes in *Bombyx mori* with holocentric centromere using genome-wide expression screening based on the knockdown of *BmSCE*, *BmESC*, *BmPHO*, or *BmSCM* gene, which represent the distinct complexes. As a result, the expressions of 29 genes were up-regulated after knocking down 4 PcG genes. Particularly, there is a significant overlap between targets of BmPho (331 out of 524) and BmScm (331 out of 532), and among these, 190 genes function as regulator factors playing important roles in development. We also found that BmPho, as well as BmScm, can interact with other Polycomb components examined in this study. Further detailed analysis revealed that the C-terminus of BmPho containing zinc finger domain is involved in the interaction between BmPho and BmScm. Moreover, the zinc finger domain in BmPho contributes to its inhibitory function and ectopic overexpression of BmScm is able to promote transcriptional repression by Gal4-Pho fusions including BmScm-interacting domain. Loss of BmPho expression causes relocalization of BmScm into the cytoplasm. Collectively, we provide evidence of a functional link between BmPho and BmScm, and propose two Polycomb-related repression mechanisms requiring only BmPho associated with BmScm or a whole set of PcG complexes.

## Introduction

Polycomb group (PcG) proteins have been well characterized as chromatin modifiers that contribute to epigenetic regulation [1]. Over past decades, the studies, from the initial identification as silencer on *Drosophila Hox* gene expression to the latest regulation on various developmental processes in vertebrates, have greatly improved our understanding on the regulatory mechanism mediated by PcG complexes and also advanced the development of epigenetics [2], [3], [4], [5], [6].

A remarkable property of PcG system is that it consists of multimeric complexes and each complex also contains a variety of components. To date, at least 15 PcG genes have been identified in *Drosophila* [7] and even much more PcG genes exist in mammalians [1]. These proteins form at least three distinct key complexes, including Polycomb repressive complex 1 (PRC1), Polycomb repressive complex 2 (PRC2), and Pleiohomeotic repressive complex (PhoRC) [8], [9], [10]. It is generally considered that PcG system involved gene regulation requires PhoRC recognition of the Polycomb responsive elements (PRE) and recruits PRC2 subsequently to induce a tri-methylation of histone H3 on lysine 27 (H3K27me3), which is also regarded as an epigenetic mark for further binding of PRC1 through the CHROMO domain of Pc protein [11], [12]. However, several studies, to some extent, have argued this hierarchical recruitment mechanism and suggested a more complicated and multidimensional model, which may provide the additional information worth exploring [1], [6], [13].

Some common approaches have been used to investigate the PcG roles, such as microarray screening upon deletions of PcG genes for genome-wide identification of PcG targets [12], [14], [15], chromatin immunoprecipitation combining sequence (ChIP-Seq) or microarray (ChIP-chip) for genome-wide mapping of PcG binding sites [14], [16], and protein-protein interaction analysis for characterizing the relationship of PcG proteins [11]. In human cells, global expression screening has identified some co-targets regulated by PRC1 and PRC2, and revealed the critical roles of PcG complexes in cell fate transition and differentiation [14]. However, PRC1 and PRC2 can also play the contrary functions in mouse hematopoietic stem cells [17]. Together, these results indicated a diverse and dynamic regulation of PcG system during development of various species.

PhoRC complex, consisting of Pho (YY1 in mammalians) and Sfmbt, is essential for PcG repression. This is because Pho possesses the unique sequence-specific DNA-binding activity among various Polycomb proteins [18]. ChIP-chip assays in *Drosophila* larvae revealed that over 50% of targeted regions were co-occupied by both Pho and Sfmbt proteins [19]. Therefore, Sfmbt is an important partner of Pho in recruiting PRC2 and PRC1 complexes to the target gene. Sfmbt protein contains several functional domains including four MBT repeats and one SAM, which can interact with other Polycomb proteins such as Pho and Scm [20]. Scm protein had strikingly similar domain architecture with Sfmbt and was considered as a PRC1 component [8]. However, the recent survey in *Drosophila* has also shown that Scm associates independently with a PRE of other PcG complexes [21].

We previously identified 13 PcG conserved genes in the silkworm, *Bombyx mori*, which is one species with holocentric chromosomes [22]. To characterize the potential targets regulated by PcG proteins, we analyzed the genome-wide expression changes after knocking down of four PcG genes representing distinct complexes in the silkworm BmN4-SID1 cells. Our results showed a very great overlap between BmPho and BmScm targets, of which 63% and 62% were co-occupied, respectively. We further used bimolecular fluorescence complementation (BiFC) and co-immunoprecipitation (Co-IP) assays to investigate the functional relevance between BmPho and BmScm, as well as their interactions with other PcG components. Our data also showed that the zinc finger domain in BmPho directly interacts with BmScm and contributes to their transcriptional repression. Furthermore, we presented the data that BmPho and BmScm are able to regulate a subset of Polycomb targets independently of other PcG components. Thus we speculated a novel probable mechanism mediated by Polycomb proteins in *Bombyx*.

## Results

### *Bombyx* PcG proteins were localized to the cell nucleus and capable of transcriptional repression

Our previous analysis has shown that PcG family proteins were also conserved in *Bombyx* as well as other insects including *Drosophila* [22]. Although the evolutionary conservation of a protein sequence may imply the functional similarity, it is worthwhile to explore the functions of Polycomb proteins in *Bombyx* because of its unique holocentric chromosome structure. So, we cloned 6 of 13 full-length cDNAs for BmPc, BmPh, BmSce, BmEsc, BmPho, and BmScm, and then tried to check their subcellular localizations and transcriptional repression activities.

We first determined the subcellular localization of PcG proteins in silkworm cells. Transient expression of PcG proteins fused to the C-terminus of Venus fluorescence protein was performed. All of the silkworm PcG proteins tested were localized in cell nuclei as multiple spots (Figure S1), consistent with the localization of PcG proteins in other species [23], [24].

**Figure 1:**
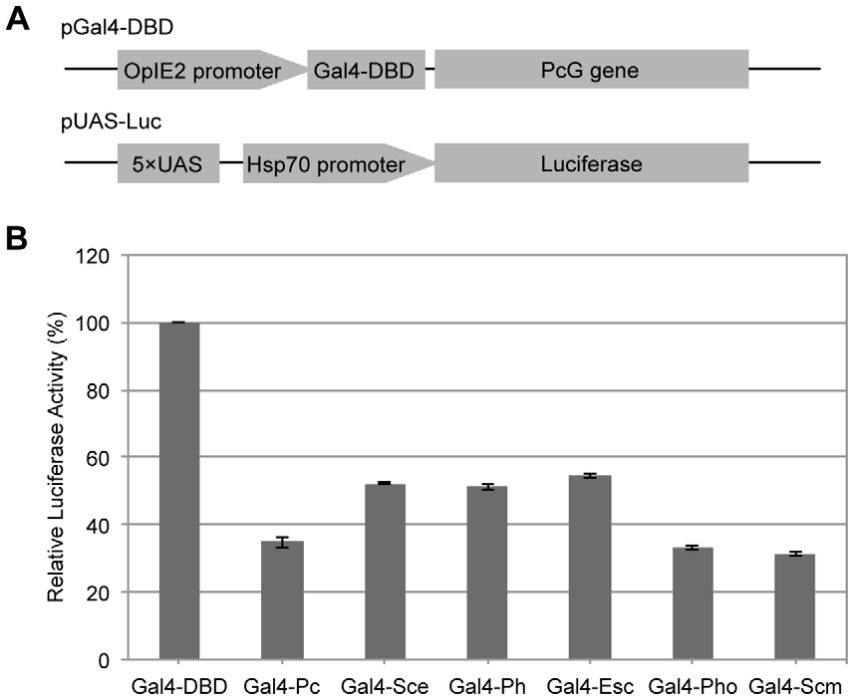
*Bombyx* PcG proteins repressed expression of the report luciferase gene. (A) Schematic of the Gal4-UAS system. The tested proteins were fused into the C-terminus of Gal4-DBD, and the luciferase gene was controlled under the silkworm HSP70 promoter with Gal4 DNA binding sites upstream. (B) Gal4-DBD fused PcG protein and luciferase report construct were co-transfected into the cells. Luciferase activity were measured after 72 h and normalized to the levels of β-galactosidase expression. The effects of PcG proteins on luciferase expression were compared with the Gal-DBD alone that averaged at 100%. Data was shown as mean ± SD of three independent experiments.

Given the crucial roles of the PcG complexes repressing the expression of the target genes [25], [26], we then examined whether the silkworm PcG proteins also function as repressors. The excellent Gal4-UAS system was constructed and used to detect their transcriptional activities (Figure 1A). We introduced Gal4-DBD fused PcG protein expression plasmids and a luciferase reporter plasmid in BmN4 cells as described in Materials and Methods. Compared with the control Gal4-DBD, the luciferase activity was significantly decreased by recruiting of various Gal4-Polycomb proteins (Figure 1B). The result also showed that the transcriptional repression capability was much higher in BmPc, BmPho, and BmScm than others.

### Knockdown of PcG genes altered the expression of different set of genes

It is known that PcG proteins regulate the expression of *Hox* genes and many other genes during development. To understand the regulatory mechanisms of PcG proteins in *Bombyx*, we sought to identify the up-regulated gene sets by knocking down of four PcG genes via dsRNA-mediated RNAi. Herein, we adopted the silkworm BmN4-SID1 cell line with an advantage of high efficiency of soaking the extracellular dsRNA [27].

**Figure 2:**
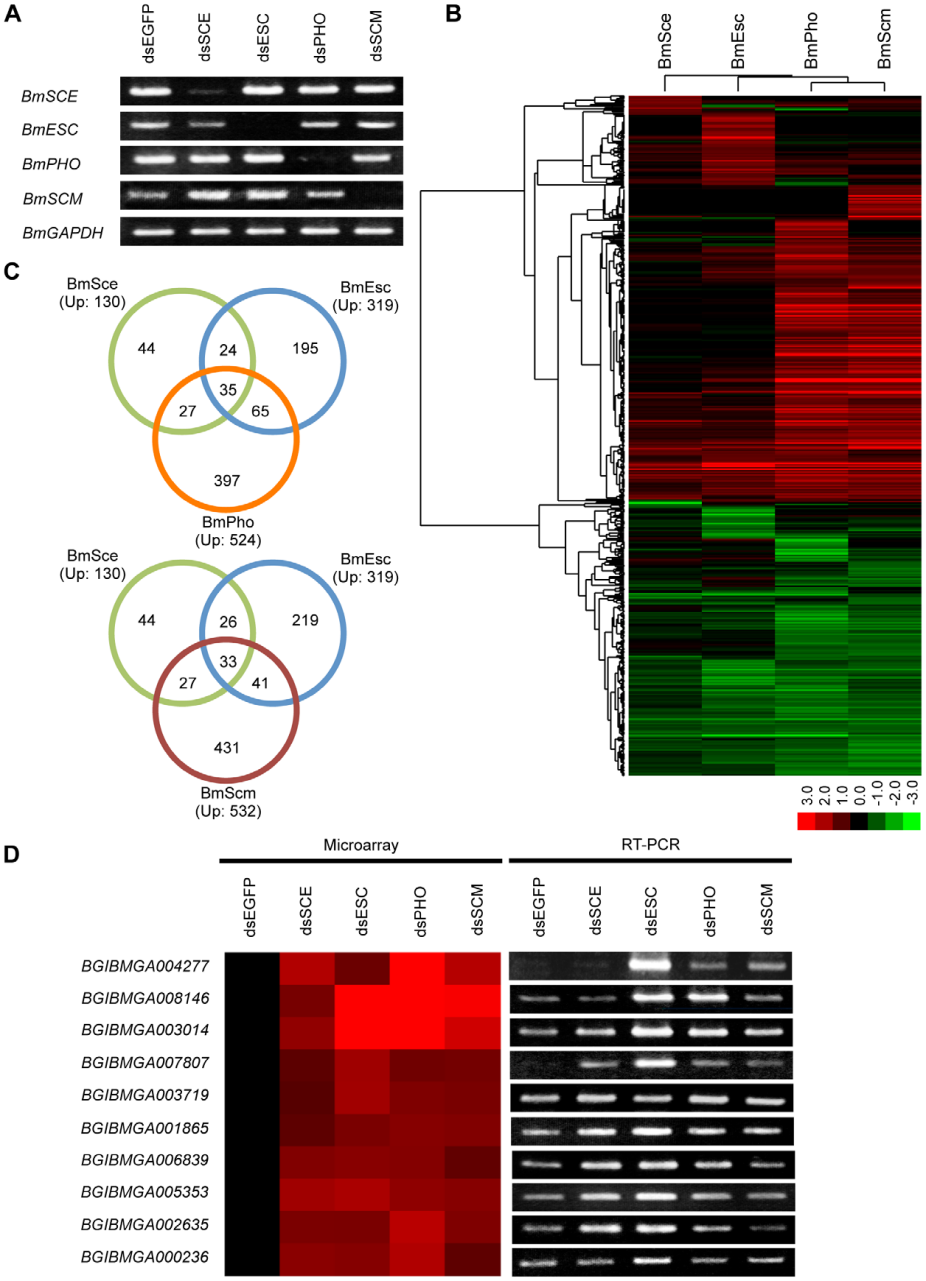
Knockdown of PcG genes in the silkworm cells resulted in altered gene expression. (A) RT-PCR analysis of the knockdown efficiency was performed from BmN4-SID1 cells 7 days after incubating with dsRNAs specific for *BmSCE*, *BmESC*, *BmPHO*, *BmSCM*, or *EGFP* (control), and the *BmGAPDH* was used as loading control for normalization. (B) Treeview diagram depicted the genes that were significantly deregulated upon PcG knockdowns. Red represented up-regulated genes, and green represented down-regulated genes in the PcG RNAi samples in comparison with the *EGFP* RNAi sample. (C) Venn diagram displayed the overlaps in genes increased >2.0-fold among BmSce, BmEsc, and BmPho (upper panel), or BmSce, BmEsc, and BmScm (lower panel). (D) Validation of gene expression changes for a selection of genes from overlap set was determined by RT-PCR. Left: expression changes from microarray data. Right: results of RT-PCR.

The specific dsRNAs for *BmSCE*, *BmESC*, *BmPHO*, *BmSCM* and control gene *EGFP* were introduced independently into BmN4-SID1 cells, and knockdown efficiency for each gene was assessed by RT-PCR experiments. The results showed that the expressions of four PcG genes were obviously attenuated after corresponding dsRNA treatment (Figure 2A). Then we used the total RNAs extracted from the same cells in RT-PCR experiments to perform a gene expression analysis using the silkworm microarray. Compared with the control of *EGFP* RNAi, we observed that the expressions of a large number of genes were changed in each PcG gene RNAi cells (Figure 2B), and defined the gene with an expression change of >2.0-fold as up-regulated gene.

**Figure 3:**
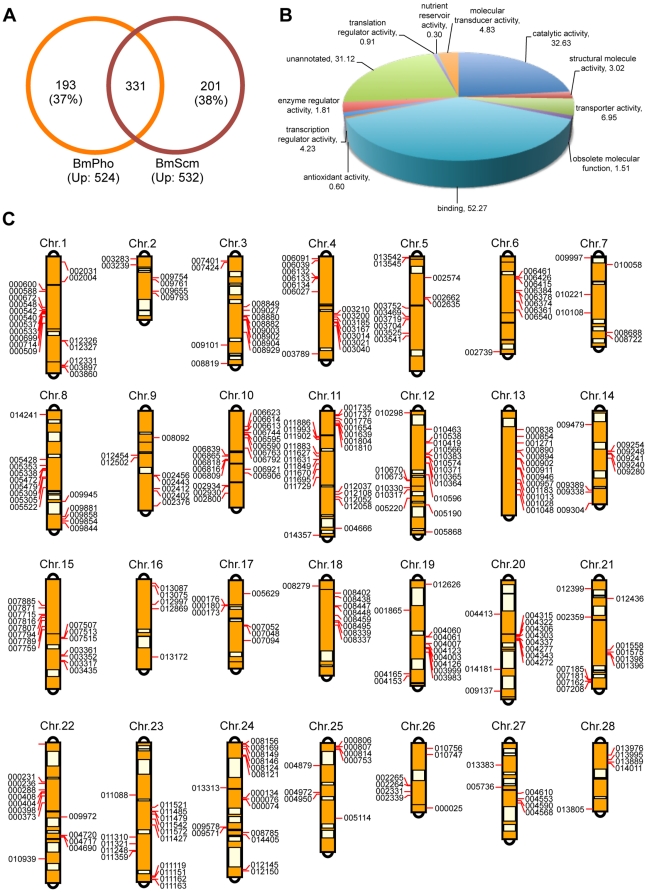
Targets of BmPho and BmScm were widely located on the silkworm genome. (A) Venn diagram showed the overlaps between BmPho and BmScm. (B) GO analysis of co-regulated genes. (C) Co-targets were widely distributed on all 28 chromosomes and most of which exhibited clustering patterns. The annotated gene name in the silkworm genome, such as BGIBMGA004277, the “BGIBMGA” was omitted in the mapping and termed as “004277”.

To identify their co-targets, we performed a Venn diagram analysis. As reported in the previous studies, Sce belongs to PRC1 complex, Esc is a PRC2 component, and Pho represents PhoRC. However, Scm has remained in confusion; the recent report showed that it could form a new complex and also recruit independently PRC1 and PRC2 complexes of Pho [21]. Thus, we first compared up-regulated gene datasets from the *BmSCE*, *BmESC*, and *BmPHO* - RNAi cells, and found that 35 genes were present and may be their common targets (Figure 2C and Table S1). Furthermore, among the datasets for *BmSCE*, *BmESC*, and *BmSCM* -RNAi cells, 33 genes were similarly up-regulated (Figure 2C and Table S2). Although only the small number of genes was co-regulated, targets of BmPho and BmScm appeared to have a significant overlap, about 63% and 62% of total up-regulated for each gene (Figure 3A and Table S3, and further analysis shown in the next section).

To validate the data from the microarray, we selected 10 co-regulated genes in the four knockdown sets (29 common targets) to perform RT-PCR analysis. As shown in Figure 2D, the expressions of all tested genes were elevated in the PcG knockdowns compared with the control of *EGFP* RNAi. Significantly, deletion of *BmESC* induced a higher expression changes than other deletions, since BmEsc protein also contributed to the tri-methylation of H3K27 as revealed in Figure S2 and our previous research [22]. These may suggest that the loss of H3K27me3 by deletion of *BmESC* would greatly release the repression of the target gene expression, and meanwhile the other components were also required for this repression. Undoubtedly, the RT-PCR results further confirmed the reliability of the expression array data.

### Targets of BmPho and BmScm were widely located on the silkworm genome

A large number of common genes were up-regulated after *BmPHO* or *BmSCM* RNAi (Figure 3A, and Table S3), suggesting that BmPho and BmScm may be cooperatively required for other PcG proteins targeting and/or direct targets regulation in silkworm cells. To gain insights into the potential roles of PcG proteins in silkworm cells, we determined the gene ontology (GO) annotations for overlapped genes from *BmPHO* or *BmSCM* RNAi. It revealed that genes extremely enriched possessed binding activity, catalytic activity, or transcription regulator activity (Figure 3B), indicating that PcG targets may play important roles in a variety of developmental processes.

We then mapped the PcG targets onto the silkworm chromosomes based on the high quality genome map and SNP linkage map. As shown in Figure 3C, PcG targets widely distributed on all 28 chromosomes and most exhibited clustering patterns. It was worth mentioning that *Hox* genes, the well-known PcG targets in other species, were not presented in our targets analysis, except for *Abdominal-B* (*BmAbd-B* annotated as BGIBMGA006384 in the silkworm genome). Based on the genomic information of silkworm *Hox* genes [28], we further checked the microarray data and found that most of the identified *Hox* genes were still up-regulated though with a slight change (lower than 2.0 folds and excluded in our analysis, data not shown). In addition, nine PcG targets could be mapped on the chromosome 6 (Figure 3C), where all silkworm *Hox* genes were also clustered [28]. Thus, we speculated that PcG proteins may be recruited to this region and also involved in the regulation of the silkworm *Hox* genes expression. However, further study needs to be done to explore this possibility.

### BmPho and BmScm could interact with other PcG components

We further examined the correlation between BmPho and BmScm in the PcG complexes formation and transcription repression, and investigated their interactions with other PcG proteins by bimolecular fluorescent complementation (BiFC) and co-immunoprecipitation (Co-IP) assays.

**Figure 4:**
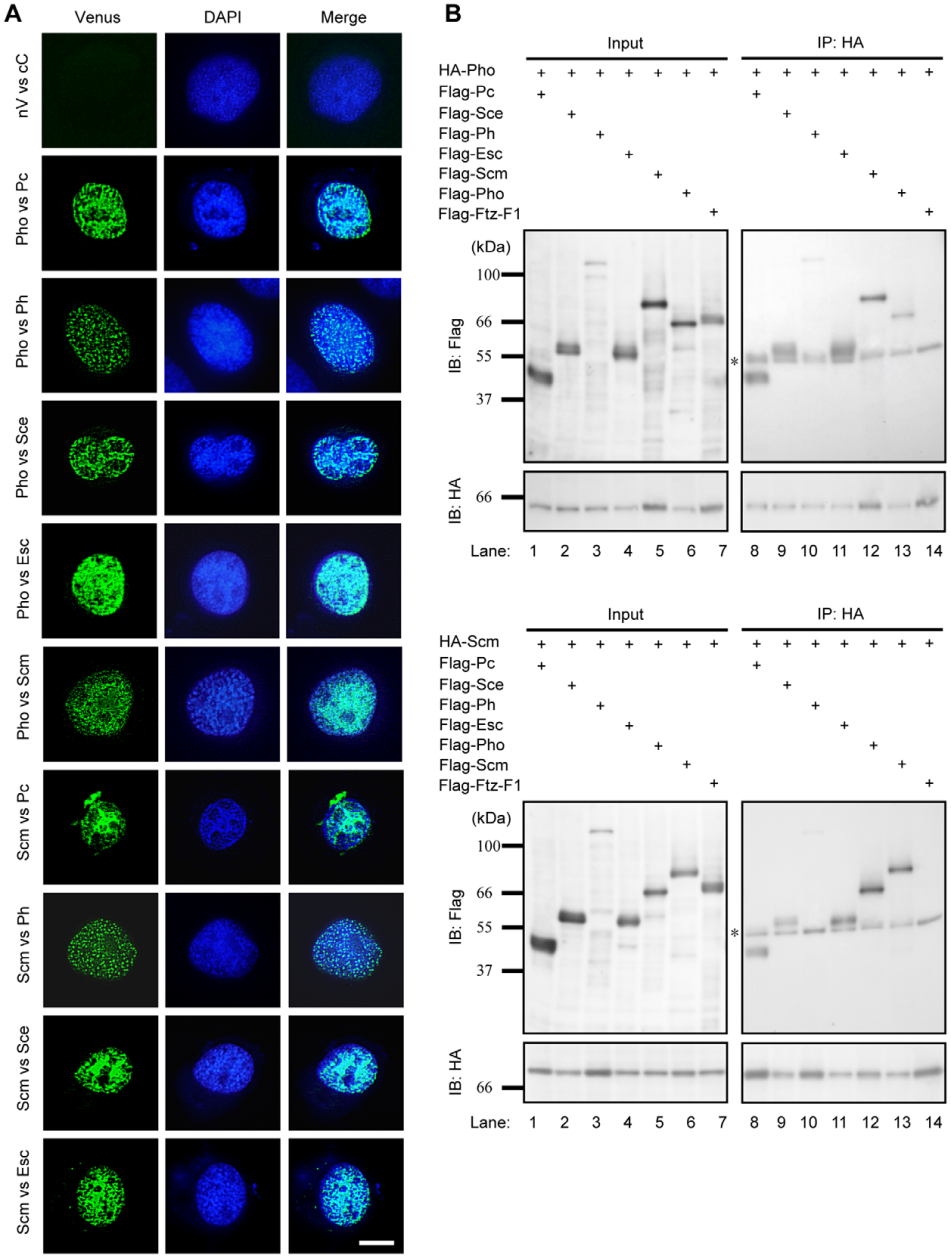
BmPho and BmScm interacted with other PcG components. (A) Interactions of different PcG proteins were visualized by BiFC analysis. BiFC complex formed through BmPho or BmScm protein with other components indicated on the left of each graph (green) and DNA stained with DAPI (blue) were imaged in silkworm cells. Scale bar: 10 µm. (B) Co-immunoprecipitation experiments further confirmed the interactions indicated in (A). As controls, Both BmPho and BmScm proteins did not interact with another silkworm nuclear hormone receptor protein BmFtz-F1 (lane 14). Asterisks represented the heavy chain of IgG.

In BiFC experiments, through the reassemble of active Venus fluorescent protein between nV-PcG and cC-PcG fusion proteins, we can monitor their interaction by checking whether BmN4 cells emit green fluorescence. As shown in Figure 4, both BmPho and BmScm could interact with BmPc, BmPh, and BmSce (PRC1 components), as well as BmEsc (PRC2 component), and also each other. Moreover, BiFC results showed distinct punctate nuclear distributions of PcG interactions, which may be the so-called “PcG bodies” previously [29]. This was consistent with the subcellular localization pattern shown in Figure S1. These observations may reflect the extensive chromatic allocation of PcG targets in the silkworm genome. Additionally, we also noted that some transfected cells presented large dots or patch-like green signals, although details of this distinct pattern remained unknown.

To verify the interactions observed above, we carried out Co-IP assay. Generally, HA-tagged BmPho or BmScm protein was co-expressed with one of Flag-tagged PcG proteins in BmN4 cells, immunoprecipitated with anti-HA antibody, and visualized by western blotting using anti-Flag antibody. Flag-BmFtz-F1 encoding a nuclear hormone receptor protein was used as a control [30] (Figure 4B). As a result, BmPho and BmScm interacted with all tested PcG proteins but not with BmFtz-F1, respectively. Interestingly, like BmPho protein, BmScm protein was also able to interact with itself, this may suggest that Polycomb proteins spread on the chromatins through bridging of BmPho homodimer and/or BmScm homodimer.

### BmPho and BmScm regulated a subset of PcG target genes expression independently of other PRC1 and PRC2 complexes

The above studies suggested a functional responsibility of BmPho and BmScm for the PcG complexes formation and their regulation on target genes. However, the significant co-targeting by BmPho with BmScm rather than with other PRC1 or PRC2 component raised the possibility that these two proteins have the ability to repress gene expression independently without the other PcG complexes.

**Figure 5:**
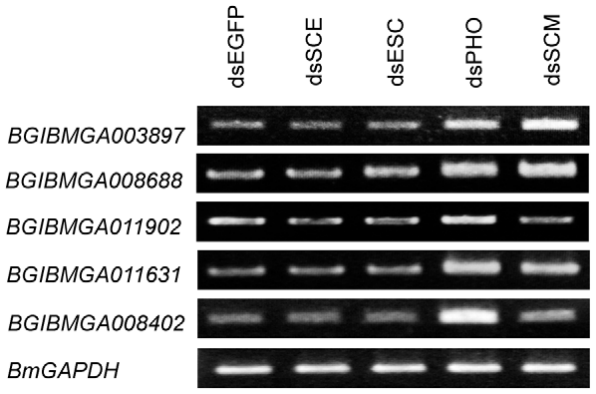
BmPho and BmScm regulated a subset of PcG target genes independently of other PcG components. RT-PCR was used to analyze the up-regulations in knockdowns by *BmPHO* and *BmSCM*, but not by *BmSCE* or *BmESC*, using the same templates from the microarray experiments, and the selections of genes were from the microarray data.

To test this possibility, we, again, selected the common genes up-regulated by *BmPHO* RNAi and *BmSCM* RNAi, but not by *BmSCE* or *BmESC* RNAi. RT-PCR analysis showed that the expression of these genes increased after depletion of *BmPHO* or *BmSCM* compared with the control (Figure 5). By contrast, the expression after knockdown of *BmSCE* or *BmESC* did not show any changes. This observation indicated that this set of genes was only derepressed in *BmPHO* and *BmSCM* RNAi but remained repressed in *BmSCE* and *BmESC* RNAi, clearly demonstrating that BmPho and BmScm can regulate a subset of PcG target genes through an unusual PcG system. This finding may also explain that the reason why there was higher inhibition activity by Gal-Pho and Gal-Scm shown in Figure 1B, and was also consistent with the result that knockdown of other Polycomb components could not ease the transcriptional repression mediated by the Gal4-Pho or Gal4-Scm (Figure S3).

### C-terminus of BmPho containing zinc finger domain was involved in the interaction between BmPho and BmScm

**Figure 6:**
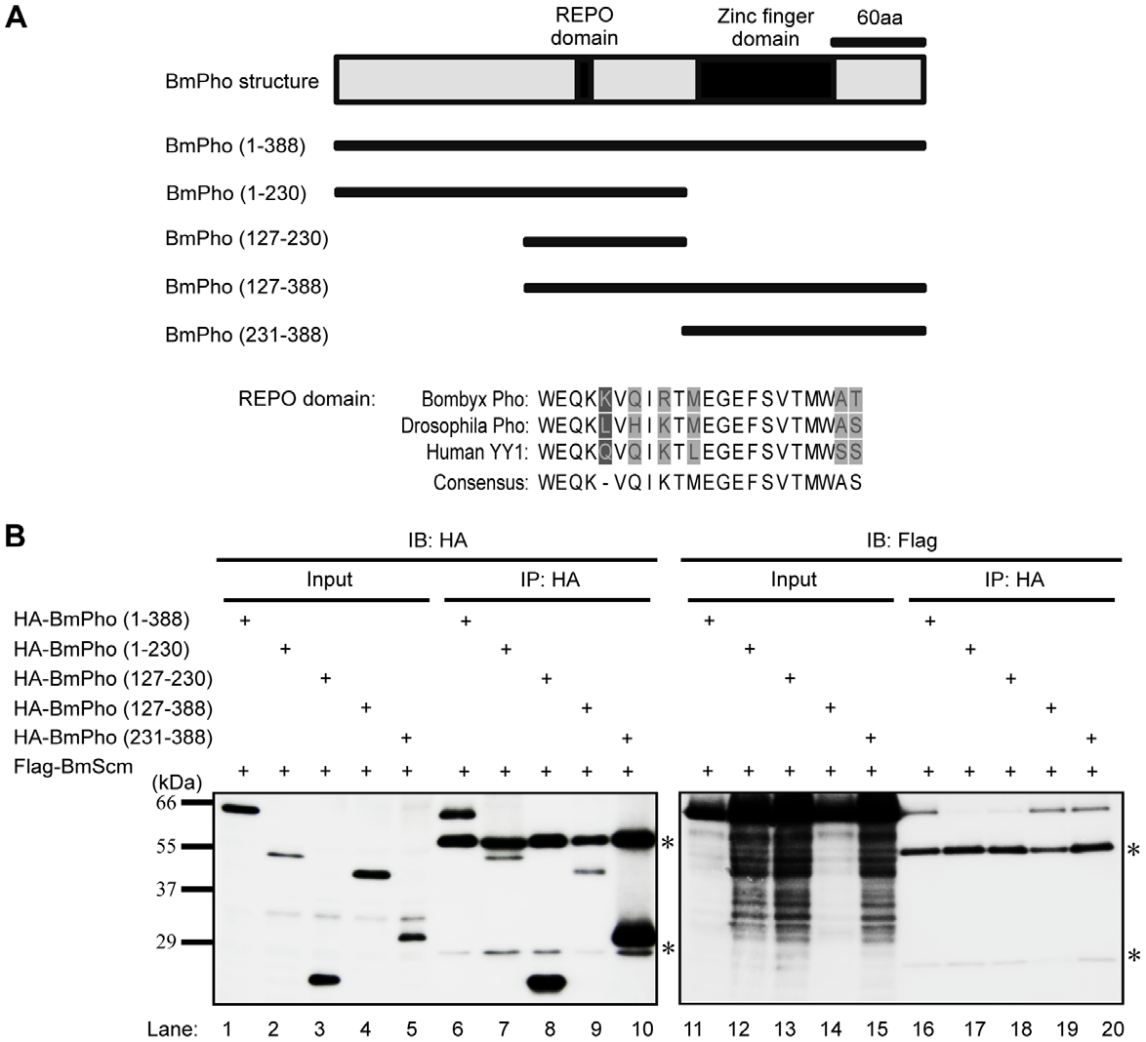
C-terminus of BmPho containing zinc finger domain was involved in the interaction between BmPho and BmScm. (A) Schematic of distinct BmPho truncates constructed in this study, and the conservation of REPO domains from *Bombyx*, *Drosophila*, and human. (B) Co-immunoprecipitation experiments were performed between various BmPho deletions and full-length of BmScm. Asterisks represented the heavy chain and light chain of IgG.

The previous studies have shown that a distinct region containing a REPO domain (conserved between Pho and mammalian ortholog YY1) of *Drosophila* Pho can efficiently interact with both Pc and Ph leading to the recruit of PRC1 complex [26]. In order to uncover the interactional mechanism between Pho and Scm in *Bombyx*, a set of deletion mutants of BmPho was constructed (Figure 6A), and was used to analyze whether REPO domain of BmPho also participates in the interaction with BmScm or how the interaction occurs between BmPho and BmScm.

The full-length of BmPho and different truncates were subjected to Co-IP with BmScm (Figure 6B). Western blotting results showed that the C-terminus of BmPho could efficiently bind to BmScm than REPO domain (Figure 6B, lane 20 and lane 18), although it should be noted that REPO domain was also conserved in *Bombyx* as well as in *Drosophila* and human (Figure 6A). This may because the REPO domain was disrupted by truncation in our construct and then lost its functional structure. We further performed a Co-IP experiment with BmPc, and found that the REPO domain of BmPho, rather than the C-terminus, could interact with BmPc (Figure S4). Together, our results demonstrated that the distinct structures in BmPho were involved in different interactions with different proteins. Additionally, the C-terminus containing four zinc fingers repeats domain was essential for DNA binding. Therefore, we concluded that the zinc finger domain in BmPho has the potential roles of protein binding as well as DNA binding.

### Zinc finger domain of BmPho was critical for the transcriptional repression

**Figure 7:**
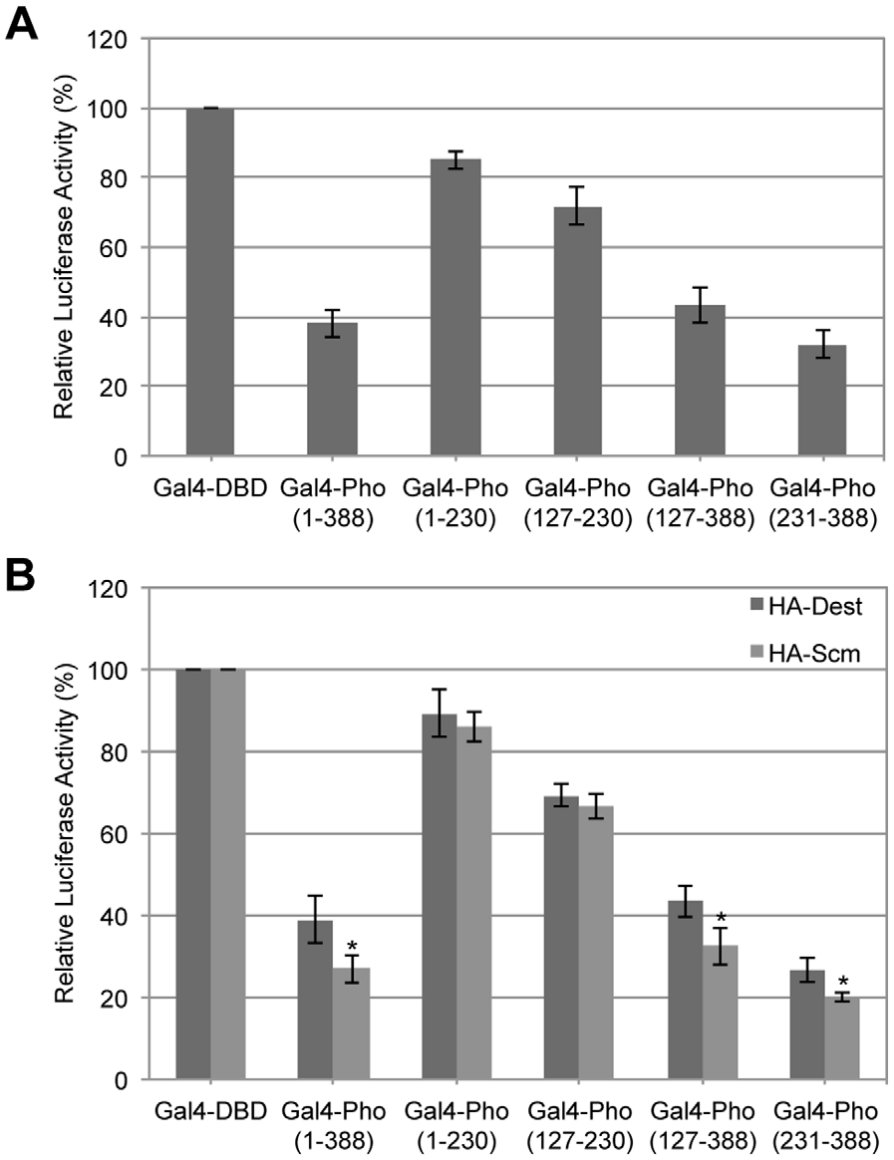
Zinc finger domain of BmPho was critical for the transcriptional repression. (A) Gal4-DBD fused BmPho deletions were used to test their transcriptional activities. The transfection method and luciferase measurement were shown in Figure 1. (B) Overexpression of BmScm promoted the transcriptional repression mediated by the interaction with zinc finger domain of BmPho. The various Gal4-DBD fusions in (A) were co-transfected either with empty vector (HA-Dest) or with BmScm expressing vector (HA-Scm). The data were compared between HA-Scm treatment and the corresponding control HA-Dest treatment by the Student's t test, *P<0.05.

The deletion mutants described above were also used to mine the specific domain that contributed to the repressive function of BmPho. When these truncates were fused to the Gal4-DBD construct and used for repression analysis, as shown in Figure 7A, the C-terminus of BmPho containing zinc finger domain possessed strong transcriptional repression activity. With difference from the previous research that the REPO domain of human YY1 was necessary for PcG repression [31], we did not find the significant inhibition activity in the region containing REPO. Also, the report mentioned that human REPO domain would lose its silencing activity to a non-PcG-sensitive reporter [31].

In order to examine whether the interaction between BmPho and BmScm could enhance the repressive activity of BmPho, we performed an experiment to overexpress BmScm protein. The result showed that, only the Gal4-Pho constructs including the zinc finger domain, the promotion of transcriptional repression by BmScm could be clearly observed (Figure 7B).

### Knockdown of *BmPHO* partially affected the localization of BmScm

**Figure 8:**
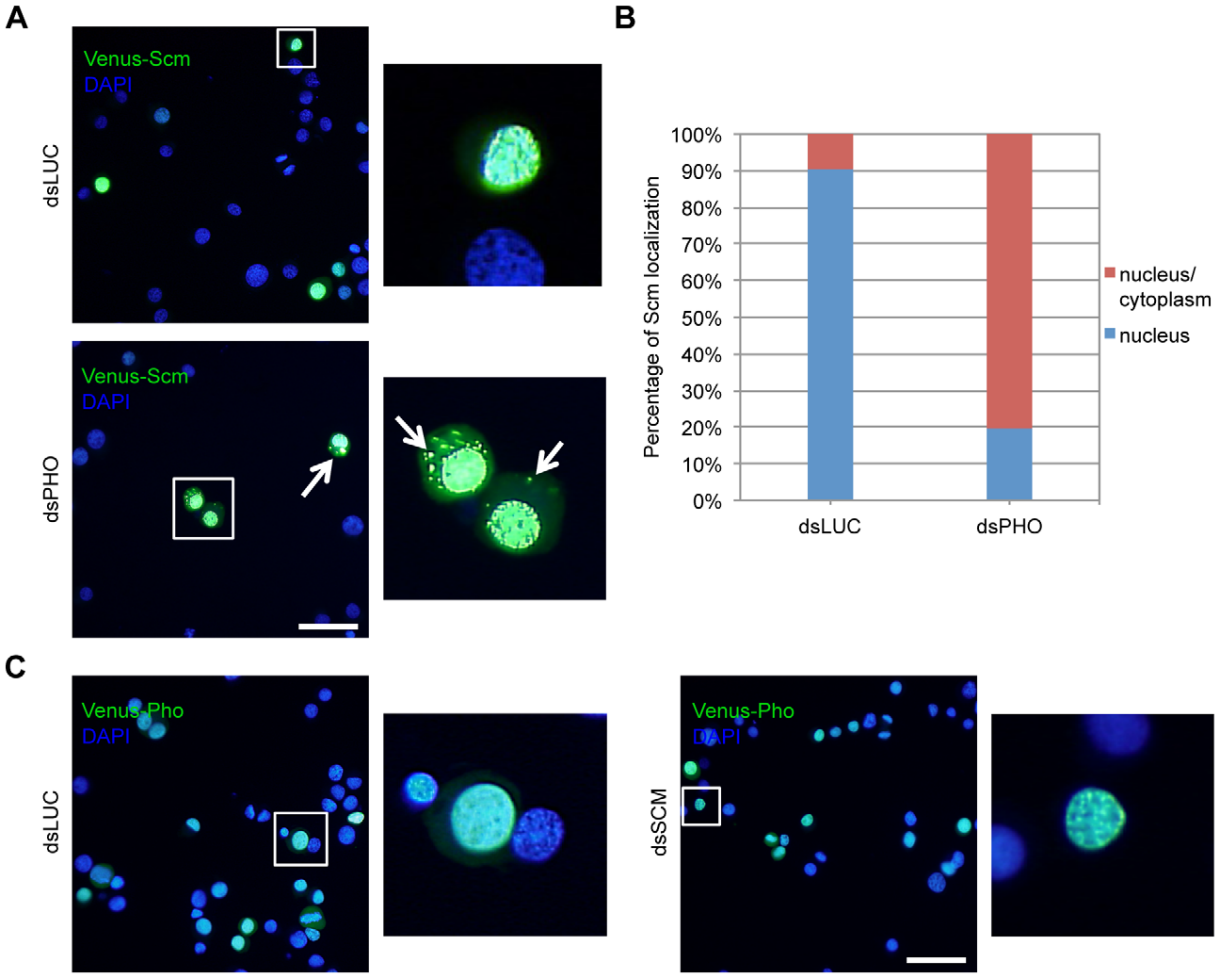
Knockdown of *BmPHO* partially affected the localization of BmScm. (A)Down-regulation of *BmPHO* greatly increased the localization of BmScm in the cytoplasm. The BmN4-SID1 cells were pre-cultured with dsRNA specific for *BmPHO* or dsRNA against *LUC* for 3 days, and then were transfected with Venus-fused BmScm for another 3 days. The cells were imaged by microscope and the nuclei DNA was counterstained with DAPI. The fluorescence signals localized in the cytoplasm were indicated with arrows. Scale bar: 50 µm. (B) The cell numbers of variant distributions indicated in (A) was counted under microscope from various fields, and the percentage of each fraction was shown in the graph. N = 96 for dsLUC treatment, and N = 77 for dsPHO treatment. (C) Down-regulation of *BmSCM* did not change the localization of BmPho. Scale bar: 50 µm.

Pho as sequence-specific DNA binding protein plays crucial roles in the targeting of PcG proteins and the subsequent repression on target gene expression [32]. In attempt to examine whether the knockdown of *BmPHO* could change the localization of other PcG components, we used the Venus-fused PcG proteins to monitor their localization in the cells treated with dsRNA against *BmPHO*. As shown in Figure 8A, loss of BmPho expression greatly affected the cellular distribution of Venus-fused BmScm. Approximately 80% of cells with signals showed both nuclear and cytoplasmic localization of Venus-BmScm, whereas less than 10% of cells in the control with dsLUC treatment presented a similar pattern (Figure 8B). This observation indicated that knockdown of the endogenous *BmPHO* may lead to the release of BmScm from the nuclei. In contrast, the down-regulation of *BmSCM* did not affect the distribution of BmPho protein (Figure 8C). Namely, BmPho was perhaps required for the recruitment of BmScm in the specific locations. However, the localizations of the other PcG proteins could not be affected significantly after *BmPHO* knockdown (Figure S5). Probably, although with slight disassociation from target loci, the knockdown of *BmPHO* was insufficient for the great changes of distributions of PRC1 and PRC2 components.

### Knockdown of *BmPHO* inhibited cell proliferation

**Figure 9:**
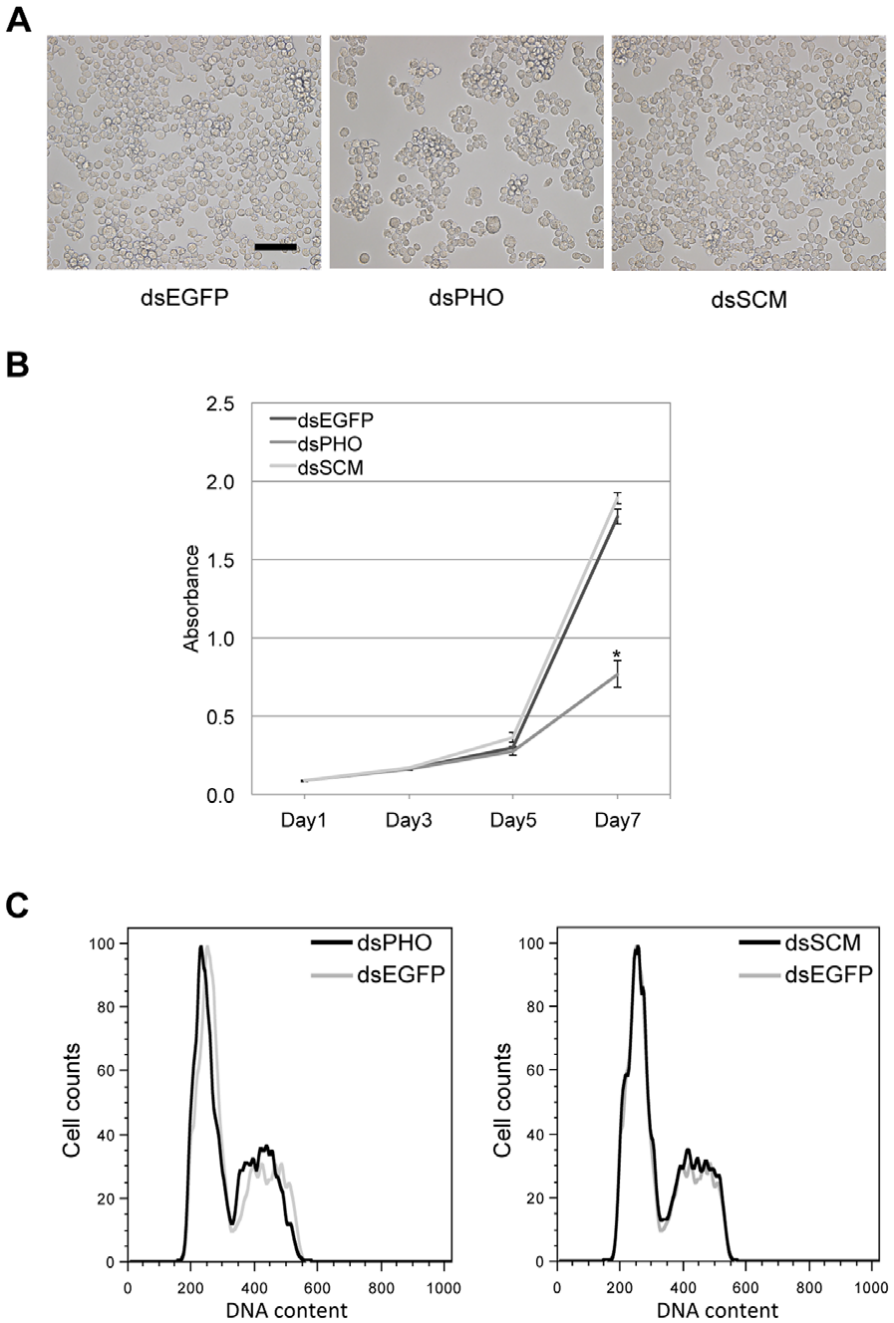
Knockdown of *BmPHO* inhibited cell proliferation. (A) Representative graphs of cells treated with dsRNAs against *EGFP*, *BmPHO*, or *BmSCM*, respectively. Scale bar: 50 µm. (B) Down-regulation of *BmPHO* suppressed cell proliferation by using WST-8 assay. *P<0.001, compared to the control of *EGFP* RNAi cells. (C) The distribution of cell cycle in dsRNAs treated cells was measured using flow cytometry. The merged results from PcG knockdown and control treatment were shown in each panel.

Mounting evidence has indicated that many PcG proteins can regulate cell cycle progression and affect proliferation by direct or indirect regulation of cell cycle-related factors [33], [34]. We wondered whether silkworm PcG proteins are also involved in cell growth regulation. Intriguingly, each cell with knockdown of each PcG gene used for microarray experiment has not shown a significant change in cell growth, with the exception of *BmPHO*. As shown in Figure 9A, following the depletion of *BmPHO*, decreases in cell numbers and increased cell compaction occurred. In fact, there was no report, to date, about the effect of *PHO* knockdown on cells. To further determine the effect of *BmPHO* on the silkworm cells, we monitored the cell growth by using WST-8 assay. Indeed, time course of cell proliferation curve showed the significant decreases in cell numbers after *BmPHO* knockdown, especially on the 7th day (Figure 9B). However, *BmPHO* down-regulation did not affect cell cycle progression, as assessed through flow cytometry assay (Figure 9C).

## Discussion

The recent genome-wide characterizations of PcG target genes have revealed the regulatory mechanisms underlying the roles of PcG proteins during development in *Drosophila* and mammals [12], [14], [35]. It is considered that the repressed expression of target gene-mediated by PcG complexes is implemented through the interactions of PcG proteins themselves and even with other chromatin-remodeling factors leading to the condensed chromatins that block the accessibility of activators [36].

Although we have understood that PcG targets is recognized and bound by Pho protein through the special DNA sequence as PREs at the target loci in *Drosophila*, it is still unclear that how the PcG proteins are recruited to the target genes in other species. Moreover, YY1, the mammalian ortholog of Pho, is not always correspondent with this fashion as revealed in *Drosophila*, and even little evidence is shown to support this model. Interestingly, the recent reports have demonstrated that long non-coding (lnc) RNAs and short RNAs all play crucial roles in the recruitment of PcG complexes in mammals [37], [38], [39].

Here, we firstly investigated the roles and signaling mechanism of PcG proteins in *Bombyx*, a Lepidoptera model insect. In this study, we have cloned and characterized 6 out of 13 PcG genes that we have previously identified [22]. All of silkworm PcG proteins studied localized in the nuclei and acted as transcriptional repressors. Then, we predicated the PcG targets through genome-wide expression screening based on PcG genes RNAi in the silkworm cells and found that a set of PcG targets was co-regulated by the all PcG proteins tested in *Bombyx*. Most of them belonged to the GO functional categories of binding activity, catalytic activity, or transcription regulator activity. This indicated a potential epigenetic mechanism on the regulation of these genes expression.

It was of great interest that BmPho often co-regulated gene expression with BmScm (Figure 3). Previous studies have shown that in *Drosophila* Pho can interact with Sfmbt and form PhoRC complex and this complex is involved in the recruitment of other complexes [18], and Scm also possesses domains similar to Sfmbt [40]. These findings promoted us to ask whether Scm and Pho could also form a complex like PhoRC. Actually, several evidences from our results supported this opinion. First, BmScm can interact with other PcG proteins, as revealed by BmPho (Figure 4), suggesting that BmScm displays the similar binding profile like BmPho. Second, the C-terminal domain including zinc finger motifs in BmPho is required for its interaction with BmScm (Figure 6) and sufficient for the transcriptional repression, which could be enhanced by interaction with ectopic overexpression of BmScm (Figure 7). Finally, the localization of BmScm partially depends on BmPho rather than BmPho localization dependent on BmScm (Figure 8), probably indicating the dependency of BmScm recruitment on BmPho protein. Together, our data may not be consistent with the report that *Drosophila* Scm binds to the chromatin locus, without the aid of other PcG proteins including Pho [21]. However, it should be noted that this report mainly focused on one PRE site of *Drosophila* Ubx. Hence, we speculated that, like the PcG protein independent binding of Scm on this locus, maybe there are several styles to form PcG-related repression complexes. Undoubtedly, this report gave us some clues to investigate the regulatory mechanism for specific gene locus-mediated by PcG system.

**Figure 10:**
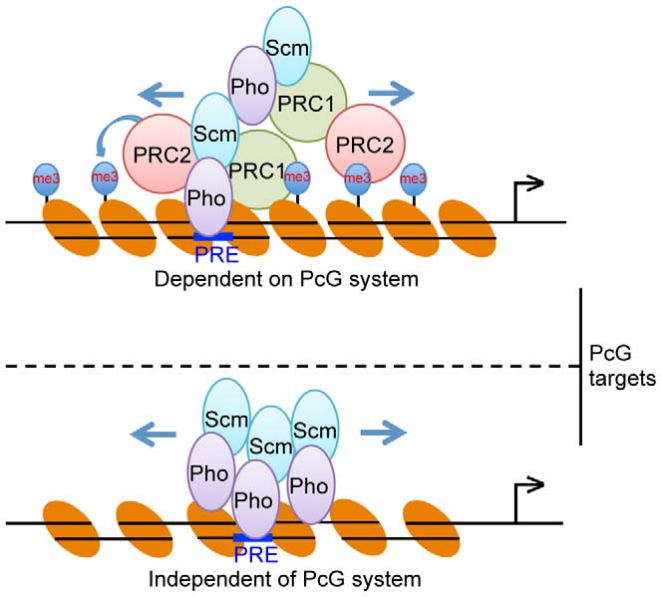
Proposed models for the regulation of PcG target gene expression-mediated by BmPho and BmScm. In this study, we envision that BmPho and BmScm regulate two classes of PcG targets. One class is based on the classic PcG system (Top), and the other one is attributed to the complex itself (Bottom). In the top panel, the DNA-binding protein BmPho associated with BmScm recognizes Polycomb response elements (PRE) and recruits other PRC2 and PRC1 complexes. PRC2 catalyzes the H3K27me3 mark and spreads out bilaterally, finally, resulting in a condensed chromatic status. In the bottom panel, the complex of BmPho and BmScm binds to PRE in the target gene, and subsequently forms the repeated units probably through homodimer and/or heterodimer themselves. Further details are shown in the text.

Importantly, we presented the potential evidence that the complex of BmPho and BmScm can regulate a group of PcG targets in a manner of independence from other PcG components (Figure 5). According to this, it may have at least two potential action models for PcG regulation in *Bombyx*. For one class of PcG targets, the entire PcG complexes are required for regulating the expression of these target genes, and BmPho and BmScm complex will be essential for the recognition and for recruiting of PRC1 and PRC2 complexes. The resulting big complexes will spread and condense the local chromatins via the tri-methylation of H3K27 and then effectively repress gene expression (Figure 10, top panel). For another class of PcG targets, BmPho and BmScm can directly regulate their expression, regardless of the presence or absence of PRC1 and/or PRC2 complexes. In this case, the formation of homodimer or heterodimer between BmPho and BmScm may be indispensable for inhibitory effects (Figure 10, bottom panel). It is not clear yet, however, how the PcG system distinguishes these aspects. Perhaps, the distinct circumstance and location for specific chromatins of one gene will be critical for providing a signal in such a condition. Hence, further investigation on specific gene should confirm this hypothesis.

In fact, the regulatory mechanism of PcG system was more complicated in mammalians. For instance, a study in mouse hematopoietic stem cells has revealed an opposite role mediated by PRC1 and PRC2 complexes based on the expression microarray analyses after mutating of different Polycomb components [17]. Thus, we also analyzed the down-regulated genes from our microarray data. Obviously, some of genes could be co-targeted by the different silkworm Polycomb proteins. Interestingly, unlike the observation in mouse cells, we cannot obtain the data of showing the opposite regulation in *Bombyx*, such as the common targets between up-regulations in PRC1 depletion and down-regulations in PRC2 depletion, as well as the contrary (data not shown). This suggested that PcG proteins in insect have somewhat different functions versus to that in mammalian and reflects the relative simple mediating mechanism in *Bombyx*. However, the future analysis in other insects, such as *Drosophila*, will give us some very meaningful clues.

Additionally, our study also showed that knockdown of endogenous *BmPHO* gene by RNAi significantly affected cell growth, but not cell cycle (Figure 9). From the microarray data, we found that the expressions of some cell cycle regulator genes, such as *BmCDC25* (BGIBMGA011902) and *BmCYCLINJ* (BGIBMGA008688), were up-regulated after *BmPHO* or *BmSCM* RNAi. RT-PCR experiment also confirmed this result (Figure 5). Although it is well known that these genes play critical roles in cell cycle progression [41], [42], the effect of *BmPHO* knockdown on cell proliferation might not be mediated by these factors. This is because that *BmSCM* knockdown has no effects on proliferation. Therefore, it may be other responsible genes that are required for the regulation of cell proliferation in *Bombyx*, and more work needs to be done to clarify the mechanism in which BmPho is involved. However, this finding indicated that the silkworm Polycomb protein BmPho could promote cell proliferation, which was yet not reported in other species.

In conclusion, the data described in this work provides some novel insights into the regulation of gene expression by Polycomb proteins. Based on this, we explored two potential models for the dynamic regulation in *Bombyx*, one of which is dependent on PcG system that requires all three complexes, whereas the other is independent of PcG system that is primarily attributed to the BmPho and BmScm complex. Both of these two models, however, require the involvement of Polycomb proteins BmPho and BmScm. This indicates that the interaction of BmPho and BmScm proteins to form a functional complex plays important roles in the targeting and regulation of Polycomb proteins-mediated transcriptional repression in *Bombyx*. It would be interesting to examine whether this case is also present in *Drosophila* or other species, which could enrich our understanding of the regulatory mechanism of Polycomb proteins.

## Materials and Methods

### Cell lines

The silkworm BmN4 cell line (a gift from Dr. Chisa Aoki, Kyushu University Graduate School) and BmN4-SID1 transgenetic cell line (stored in our laboratory) [27] were maintained at 27°C in IPL-41 medium (Sigma) supplemented with 10% fetal bovine serum (FBS) (Gibco).

### DNA constructs

Based on our previous *in silico* identification of the silkworm PcG genes [22], we isolated 6 clones representing the full-length cDNAs encoding for BmPc, BmPh, BmSce, BmEsc, BmPho, and BmScm proteins (GenBank accession number: AB607839, AB607840, AB607836, AB607838, AB607837, and AB607835) using cDNA from BmN4 cells as a template, and the primers used were listed in Table S4. These clones were further inserted into a pENTR™11 (Invitrogen) vector to construct 6 entry plasmids accordingly. The nucleotide sequences of plasmids were confirmed by DNA sequencing.

To construct the reporter genes or fusion expression vectors used in this study, destination vectors including pi2VW, pnVW, pcCW, pi2FW, pi2HW, and pGal4-DBD constructed in our laboratory (details of vector information are available upon request) [43] were used for the gateway reaction.

For the deletion analyses of BmPho, different length of DNA fragments were amplified using the serial primers in Table S4 and subcloned into the entry vector, and the expression vectors were obtained as described above.

### Transient transfection

All transient transfections were carried out in 24-well or 6-well plates. The day before transfection, cells were plated at a density of 0.5×10^5^ or 2.0×10^5^ cells per well. The lipid-DNA complex preparation and transfection program were performed as described previously [43]. Cells were harvested 72 h after transfection for localization, luciferase, or immunoprecipitation analyses.

The transfection efficiency among the dishes was measured by co-transfecting the pEXP38-βgalΔIE-1 vector expressing a β-galactosidase, and the β-galactosidase activity was used to normalize luciferase activity data [44]. All experiments were performed at least three independent transfections and data were shown as mean ± standard deviation (SD).

### Subcellular localization assay

For subcellular localization analysis, 100 ng of expression plasmids for Venus fused Polycomb proteins were transfected into BmN4 cells, respectively. 72 h post-transfection, the cells were seeded on a cover slip coated with poly-L-lysine, and then fixed with 3.7% formaldehyde in phosphate-buffered saline (PBS) for 10 min and permeabilized with 0.1% Triton X-100 in PBS for 5 min. The DNA was stained with DAPI (Invitrogen).

### Transcription inhibition assay

BmN4 cells were co-transfected with 100 ng Gal4-DBD fused Polycomb proteins and UAS-Luc reporter plasmids [43]. After 72 h transfection, the cells were harvested and lysed with the lysis buffer (25 mM Tris-phosphate, pH 7.8, 2 mM DTT, 2 mM Trans-1, 2-diaminocyclohexane-N,N,N′,N′-tetraacetic acid monohydrate, 10% glycerol, 1% Triton X-100). Luciferase activity was determined according to the previous method.

### RNA interference

The synthesis of double stranded RNAs (dsRNAs) for *EGFP*, *LUC*, *BmSCE*, *BmESC*, *BmPHO*, *BmSCM* and the treatment of BmN4-SID1 cells were done according to our previous protocol [22].

### DNA microarray assay

BmN4-SID1 cells cultured in IPL-41 medium with additions of different dsRNAs were harvested after 7 days incubation. For each treatment, samples were prepared from three independent experiments and were pooled into one sample to reduce the experimental variation. Total RNA was then isolated using Trizol reagent (Invitrogen). 1 µg RNA from each sample was subjected to reverse transcription using the ReveTra Ace cDNA synthesis kit according to the manufacturer's instructions (TOYOBO). The knockdown efficiency for each gene was evaluated by semi-quantitative reverse transcription-polymerase chain reaction (RT-PCR) using gene-specific primers (Table S4) and the silkworm *glyceraldehyde-3-phosphate dehydrogenase* (*BmGAPDH*) gene was used as an endogenous control.

For microarray experiment, the hybridization and data acquisition were carried out by CapitalBio Corp. We further analyzed the data according to the previous strategy [45]. Compared the PcG RNAi samples with the control *EGFP* RNAi sample, the fold changes >2.0 were defined as up-regulated gene. We then isolated four up-regulated gene sets representing BmSce, BmEsc, BmPho, and BmScm targets, respectively. The common targets were analyzed by the Venn diagram generator on the website (http://www.pangloss.com/seidel/Protocols/venn.cgi). Hierarchical clustering was performed using average linkage under the default settings by Cluster_Treeview software from Stanford University and the control dsRNA treatment was used as a baseline expression for comparison with the PcG dsRNA-treated samples. GO functional annotations were analyzed on the website (http://silkworm.swu.edu.cn/cgi-bin/wego/index.pl) and genomic distributions were obtained by comparison with the silkworm whole genome sequence (http://silkworm.swu.edu.cn/silkdb/). All microarray data presented in this study have been deposited in the GEO database under accession number of GSE34246.

For validation of the microarray data, we randomly selected 10 common up-regulated genes and designed their primers (Table S4). Semi-quantitative RT-PCR was performed using the same cDNA templates described above.

### Bimolecular fluorescence complementation assay

Bimolecular fluorescence complementation (BiFC) analysis was based on the reassembling into a functional fluorescent protein though the association of protein fragments fused to the proteins of interest (Mon *et al.*, manuscript in preparation). 100 ng of each expression plasmid for different PcG proteins fused to pnVW or pcCW, respectively, were co-transfected into the BmN4 cells and fluorescence was observed using the same treatment protocol described above.

### Co-immunoprecipitation assay

Co-immunoprecipitation (Co-IP) was performed as described previously [43] with the minor modification: the harvested cells were lysed in RIPA buffer (50 mM Tris-HCl, pH 8.0, 150 mM NaCl, 1% Nonidet P-40, 0.5% Sodium deoxycholate, 0.1% SDS) supplemented with protease inhibitor (Complete, EDTA-free, Roche). The lysates were immunoprecipitated by using anti-HA antibody (sc-7392, Santa cruz biotechnology). The HA-tagged proteins were eluted in RIPA buffer containing 2.0 µg/ml HA peptide. The Flag-tagged proteins in the eluted protein complex were detected by immunoblotting using anti-Flag antibody (F3165, Sigma).

### Cell proliferation assay

For cell proliferation assay, 3.0×10^3^ BmN4-SID1 cells were seeded in 96-well plates and cultured in a final volume of 100 µL. dsRNA for *EGFP*, *BmPHO*, or *BmSCM* were added into the medium with a final concentration of 0.5 µg/mL. The cells were labeled with 10 µL WST-8 solution (Cell counting Kit-8; Dojindo) for 12 h before the indicated time points, such as 1st day, 3rd day, 5th day, and 7th day. The absorbance was measured at 450 nm in a 96-well spectrophotometric plate reader according to the manufacturer's protocol, and the proliferation curves were plotted using the absorbance at each time point. All of the experiments were performed in triplicate.

### Flow cytometry assay

To analyze the effect on cell cycle after knockdown of PcG genes, cell cycle distributions were determined by measuring the cellular DNA content using flow cytometry according to the previous procedure [27].

### Cell imaging

Light and fluorescence microscopy images were captured using Biozero BZ-8000 microscope (KEYENCE).

### Statistical analysis

Statistical significance of difference between the treated and the corresponding control was evaluated by the Student's t test, and a P-value<0.05 was considered statistically significant.

## Supporting Information

Figure S1 ***Bombyx*** **PcG proteins had a distinct punctate nuclear distribution in BmN4 cells.** Subcellular localization of transiently expressed Venus-PcG fusion proteins in silkworm cells was determined by fluorescence (green) and the nuclei DNA was counterstained with DAPI (blue). As a comparison, the localization of parental construct Venus-Dest was evenly expressed both in the cytoplasm and nucleus. Scale bar: 10 µm.
(TIF)

Figure S2 **Changes of H3K27me3 levels upon knockdown of** ***BmSCE*** **,** ***BmESC*** **,** ***BmPHO*** **, or** ***BmSCM*** **.** Western blotting was performed to analyze H3K27me3 levels in the PcG-depleted cells according to our previous procedure [22]. Antibody against H3 was used as a loading control.
(TIF)

Figure S3 **Knockdown of other Polycomb components** ***BmPC*** **,** ***BmSCE*** **, or** ***BmESC*** **could not exclude the transcriptional repression mediated by the Gal4-Pho or Gal4-Scm.** The BmN4-SID1 cells were pre-cultured with different dsRNAs for 3 days, and then were transfected with Gal-Pho or Gal4-Scm plasmid according to the Figure 1.
(TIF)

Figure S4 **Interaction between BmPc and distinct BmPho truncates.** Co-immunoprecipitation was carried out between various BmPho truncates and full-length of BmPc. The cell lysates were immunoprecipitated by using anti-Flag antibody and the eluted protein complex was detected by immunoblotting using anti-HA antibody. Asterisks represented the heavy chain and light chain of IgG.
(TIF)

Figure S5 **Knockdown of** ***BmPHO*** **did not significantly affect the localization of BmPc, BmPh, BmSce, and BmEsc.** The treatment and observation were according to the Figure 8 in the *BmPHO* RNAi cells. Scale bar: 50 µm.
(TIF)

Table S1 **List of common targets up-regulated by BmSce, BmEsc, and BmPho.**
(XLS)

Table S2 **List of common targets up-regulated by BmSce, BmEsc, and BmScm.**
(XLS)

Table S3 **List of common targets up-regulated by BmPho, and BmScm.**
(XLS)

Table S4 **List of primers used in this study.**
(XLS)
