# Supplementary figures and images for: Genome-Wide Identification of Polycomb Target Genes Reveals a Functional Association of Pho with Scm in Bombyx mori
Source: PLoS One. 2012 Apr 2;7(4):e34330. doi: 10.1371/journal.pone.0034330 (PMC3317521; doi:10.1371/journal.pone.0034330)

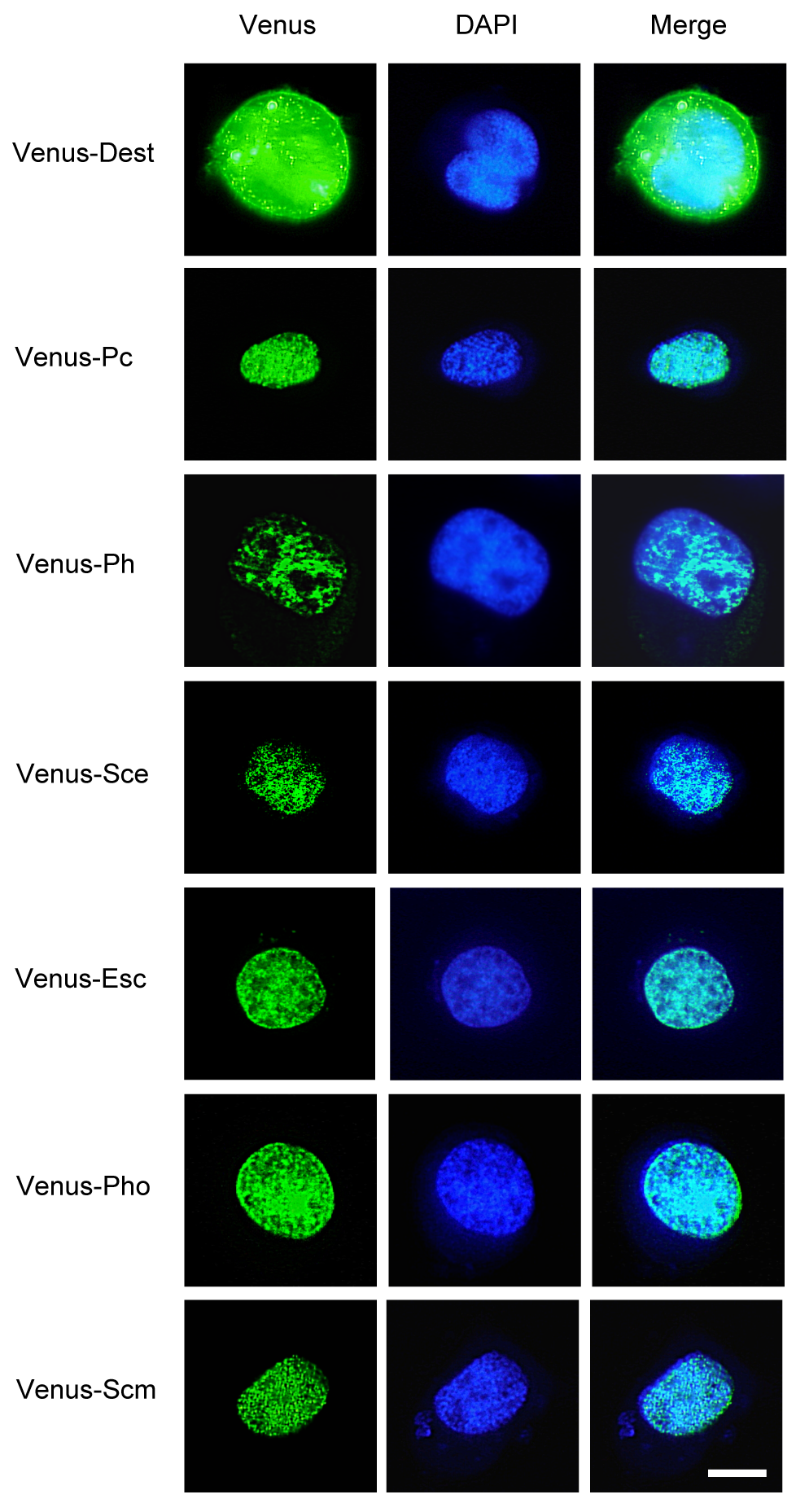

Supplement: Figure S1 — Bombyx PcG proteins had a distinct punctate nuclear distribution in BmN4 cells. Subcellular localization of transiently expressed Venus-PcG fusion proteins in silkworm cells was determined by fluorescence (green) and the nuclei DNA was counterstained with DAPI (blue). As a comparison, the localization of parental construct Venus-Dest was evenly expressed both in the cytoplasm and nucleus. Scale bar: 10 µm. (TIF) [file pone.0034330.s001.tif]

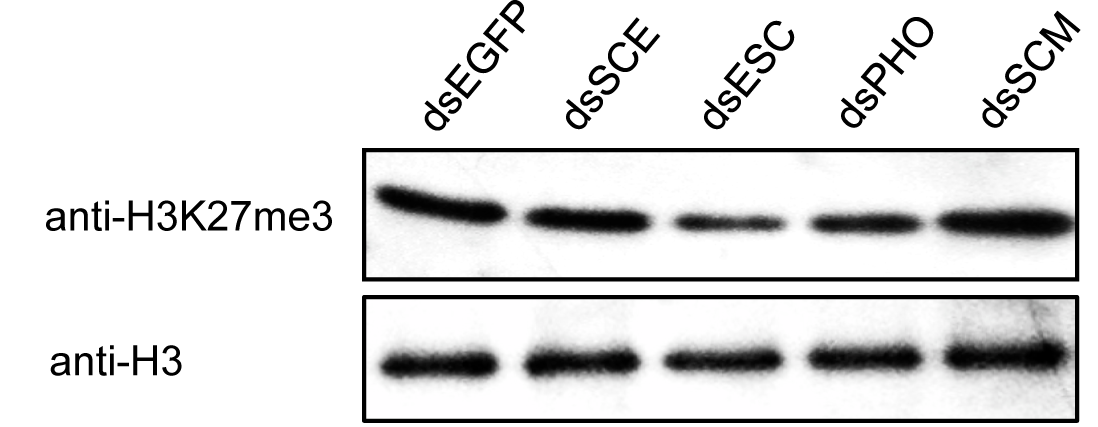

Supplement: Figure S2 — Changes of H3K27me3 levels upon knockdown of BmSCE , BmESC , BmPHO , or BmSCM . Western blotting was performed to analyze H3K27me3 levels in the PcG-depleted cells according to our previous procedure [22]. Antibody against H3 was used as a loading control. (TIF) [file pone.0034330.s002.tif]

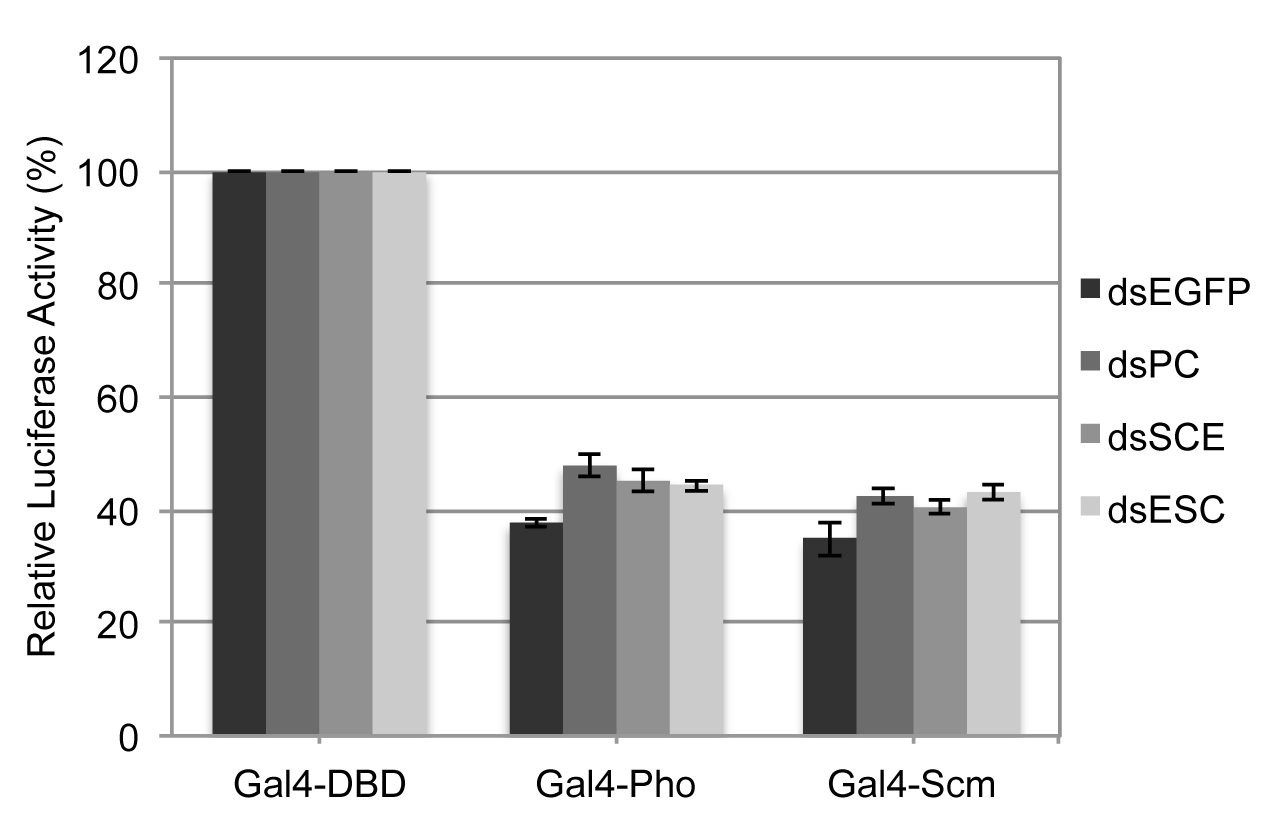

Supplement: Figure S3 — Knockdown of other Polycomb components BmPC , BmSCE , or BmESC could not exclude the transcriptional repression mediated by the Gal4-Pho or Gal4-Scm. The BmN4-SID1 cells were pre-cultured with different dsRNAs for 3 days, and then were transfected with Gal-Pho or Gal4-Scm plasmid according to the Figure 1. (TIF) [file pone.0034330.s003.tif]

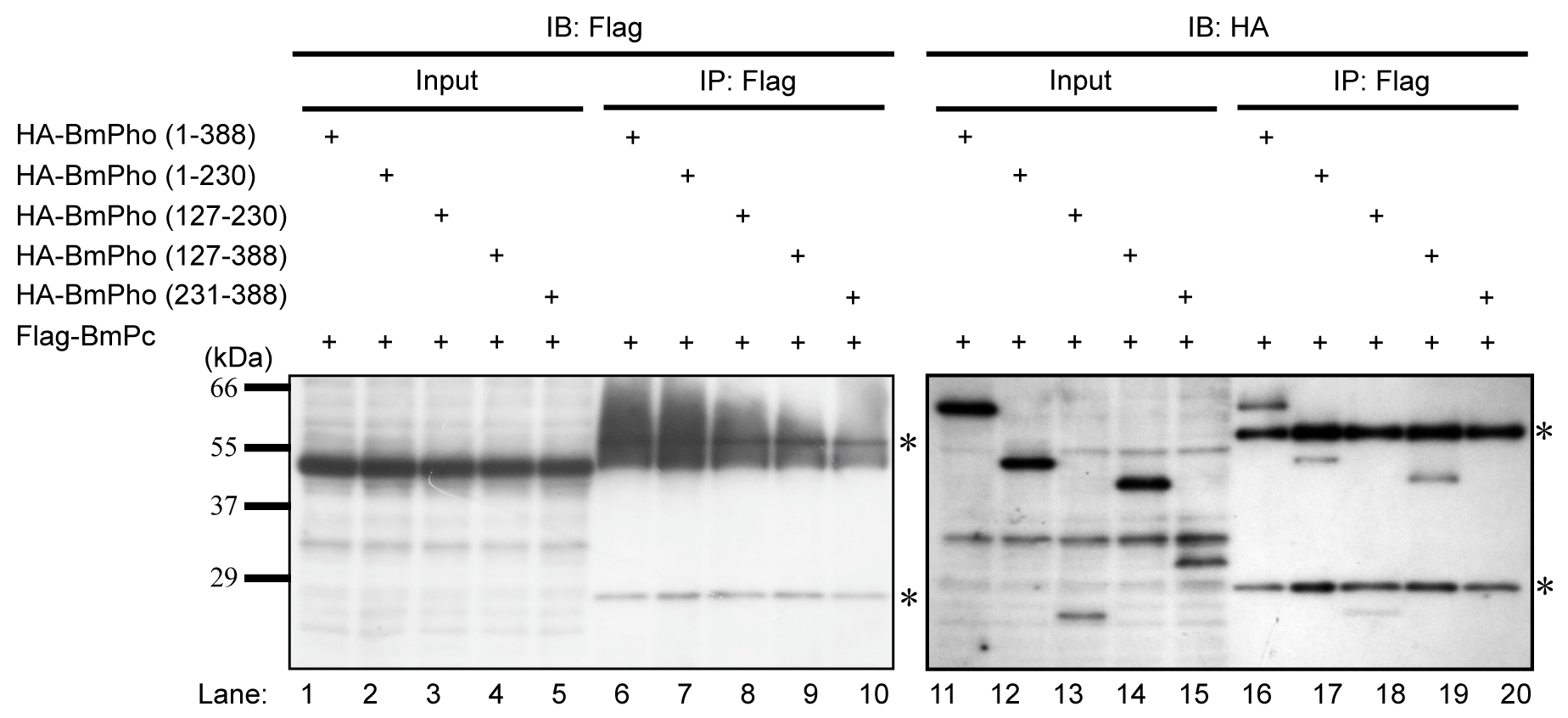

Supplement: Figure S4 — Interaction between BmPc and distinct BmPho truncates. Co-immunoprecipitation was carried out between various BmPho truncates and full-length of BmPc. The cell lysates were immunoprecipitated by using anti-Flag antibody and the eluted protein complex was detected by immunoblotting using anti-HA antibody. Asterisks represented the heavy chain and light chain of IgG. (TIF) [file pone.0034330.s004.tif]

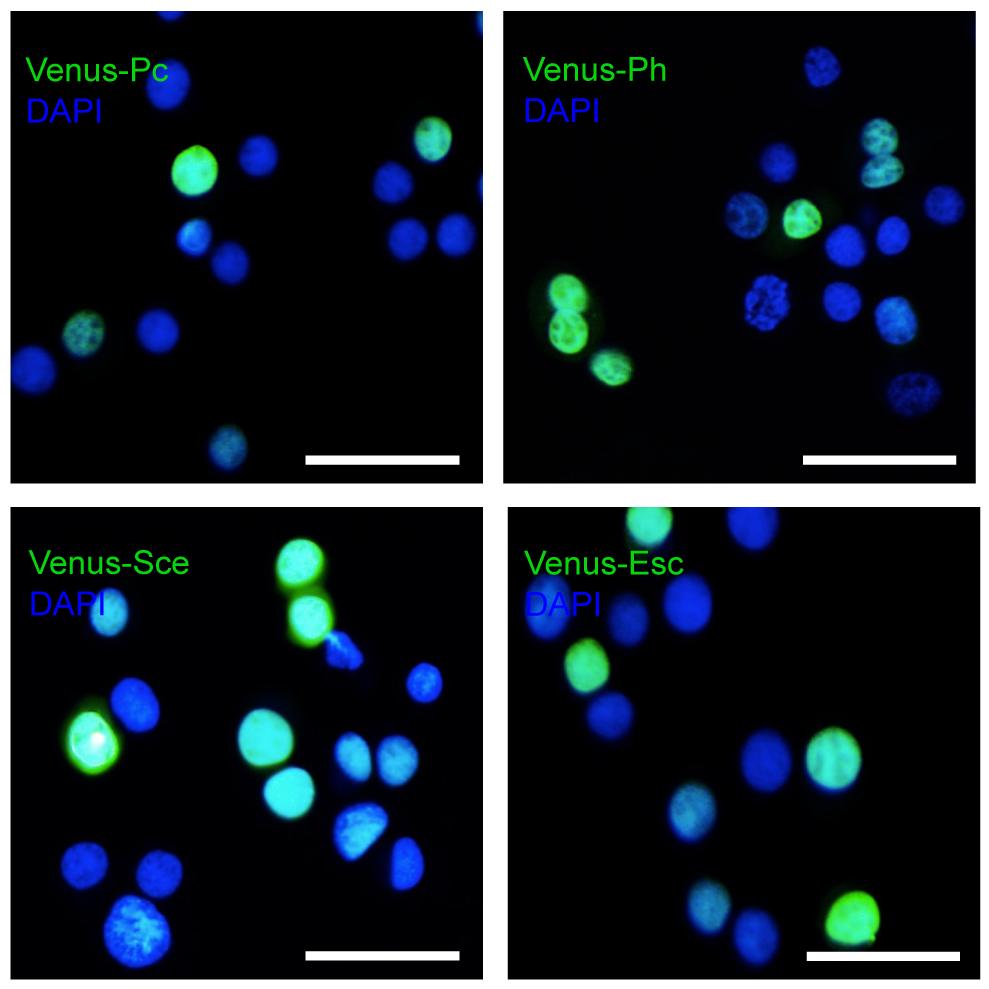

Supplement: Figure S5 — Knockdown of BmPHO did not significantly affect the localization of BmPc, BmPh, BmSce, and BmEsc. The treatment and observation were according to the Figure 8 in the BmPHO RNAi cells. Scale bar: 50 µm. (TIF) [file pone.0034330.s005.tif]
